# Modeling of the Aorta: Complexities and Inadequacies

**DOI:** 10.1055/s-0040-1715588

**Published:** 2020-12-11

**Authors:** Kumbakonam R. Rajagopal, Keshava Rajagopal

**Affiliations:** 1 Department of Mechanical Engineering, Texas A&M University, College Station, Texas; 2 Department of Clinical Sciences, University of Houston College of Medicine, Houston, Texas

**Keywords:** aorta, viscoelasticity, anisotropy, inhomogeneity, mixture, fluid–solid interaction

## Abstract

The aorta is a very complex organ comprising three layers, consisting of four kinds of tissues. It is an anisotropic, inhomogeneous, multiconstituent, and living organ that presents both a formidable challenge and a tremendous opportunity to a modeler to mathematically characterize its structure. Unfortunately, even the most sophisticated models in vogue do not faithfully take into consideration its various complexities, falling very short of putting into place a reasonable model, as they ignore many of the quintessential features that need to be taken into account. In this article, we address the various features that need to be taken into account to develop a meaningful model of the aorta.

## Introduction

Theories, among other attributes, must accomplish two tasks: (1) describe and explain what has occurred, and (2) predict that which has not yet been observed. The latter goal is even more practically important in cardiovascular medicine and surgery, as it may guide decision-making (strategy) and therapeutic conduct (tactics). The clinical goal of developing models that describe and explain the functions and responses of components of the cardiovascular system is to be able to make decisions as to medical, interventional, or surgical therapies, and to deploy specific therapies in a tailor-made fashion if possible. Such decisions may be preemptive or corrective. Aortic diseases, in particular, are consequences of the intricate interplay between biochemical reactions along the inner surface of the aortic wall and flowing blood (models of individual reactions of coagulation pathways, as well as sets of reactions and feedback loops, but with scant attention to the mechanics, emerged approximately 30 years ago, 1 ) deformation of the walls of the aorta, how blood flowing through the aorta interacts with the walls. (The seminal work of Poiseuille, a physician, concerning the flow of fluids in pipes of small diameters influenced by his interest in understanding the flow of blood, is yet used widely in engineering 2 3 4 5 6 ) influences this, and so on. Thus, the study of just biochemical reactions or just solid and/or fluid mechanics relevant to the cardiovascular system would be at best fragmentary and incomplete, and relying on such information would be inapplicable and not useful. Such a situation notwithstanding, given the complexity, intricacy, and largeness of the complete problem that requires taking into consideration biochemistry and mechanics simultaneously, we have no recourse but to simplify the problem of modeling the aorta and applying it to diseases, by taking into consideration, the quintessential features of the problems.

Accordingly, our aim here is modest; we are interested merely in delineating all the *mechanical* issues of which we need to take cognizance, to develop a robust mechanical model of the cardiovascular system that has to be melded together subsequently with the relevant biochemistry.

Describing the mechanical behavior of the aorta and even smaller blood vessels is a 3-fold model as follows: (1) modeling of the walls of blood vessels, (2) modeling the constitutive nature of blood that is flowing in these vessels, and (3) describing interactions between the walls of the blood vessel and the flowing blood. To date, all attempts at modeling the behavior of the aorta, in particular, and blood vessels in general, are profoundly incomplete and inadequate, in that they are not faithful in describing any of the three above-mentioned areas. These inaccuracies relate to mischaracterization of the following: (1) the material properties of arterial walls; (2) the structure and architecture of blood flowing both through and within (i.e., in the vasa vasorum) arteries; (3) the complex structure of blood, within which chemical reactions occur in the context of blood flow; and (4) the complex interactions between the arterial walls and flowing blood. While blood flowing in the aorta and other large blood vessels can be modeled by what is termed the Navier–Stokes constitutive relation, the models used to describe flow in smaller vessels and interactions between the arterial walls and flowing blood lack fidelity. However, the aim of this work is not to impugn earlier attempts at modeling. The intent of this paper is to outline the various factors that need to be taken into consideration in developing a more comprehensive constitutive expression for describing the behavior of arteries in the context of intraluminal blood flow. The development of a constitutive expression that takes into consideration all the aspects that will be outlined in what follows is far from realizable in short order; it must be put into place gradually.

**1. The main features of the wall of the aorta and other blood vessels that need to be considered in the development of a constitutive expression for describing their behavior are as follows:**

**Fig. 1:**
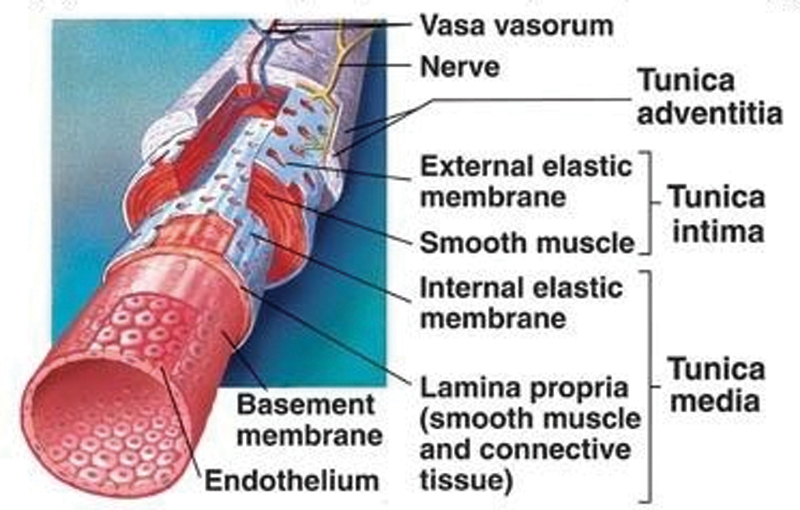
Structure of a blood vessel (Original image from Cinnamon VanPutte, Jennifer Regan, and Andrew Russo. Seeley’s essentials of anatomy & physiology. McGraw-Hill, 2015. Source: Modified image from http://www.infobarrel.com/Media/Dynamic_Structure_of_Blood_Vessel_Walls ).

- The aorta is a trilayered annulus whose thickness and cross-section vary along its curvilinear axis. By “annulus,” we are not referring to the aortic annulus, but rather, what we specifically mean is the geometric definition: a hollow tube with some defined wall thickness. The exact nature of the thickness of the layers, the variation of the cross-section, etc., are patient-specific information. In addition to the layers being comprised of materials with different structure, even within the intima, media, and adventitia, there is considerable variation, and this is also patientspecific. Thus, unlike inert matter, one cannot specify with any generality the inhomogeneous nature of a large class of biological materials.
- The walls of the aorta and blood vessels have a degree of porosity. The permeability of the aortic wall is once again patient-specific. Also, since the walls are generally undergoing large deformations, simple correlations, such as Fick's equation and Darcy's equation, and their generalizations are inappropriate to describe the flows within such porous media.
- The aortic walls are infused with blood-carrying nutrients in the vasa vasorum. As the vasa vasoram are relatively small, blood flowing within them will exhibit non-Newtonian characteristics ( Fig. 1 ).
- The aorta and other large arteries undergo large deformations. Thus, one needs to use a nonlinear measure of strain to describe the deformation, since the linearized strain can only describe small strains.
- The aorta and other blood vessels are anisotropic. Anisotropy refers to the response at a point being different along different directions. In general, in the aorta, the nature of the anisotropy varies from point to point. This variation cannot be determined, as one cannot perform experiments relevant to each point in an artery. One usually assesses anisotropy based on the global response of a piece of tissue excised from the blood vessel in question, or the anisotropy as a consequence of the microstructural features inferred on the basis of microscopy. Neither of these methodologies can capture the pointwise variation in anisotropy. More importantly, unlike crystalline material, whose microstructure can be determined by microscopy, and hence the anisotropy delineated with some degree of surety, one cannot characterize the anisotropy of biological tissues with sufficient degree of surety based on microscopic determination. Rather, one has to be satisfied by characterizations that are consequence of the measurement of global response.
- Blood flowing in very small vessels, such the vasa vasorum, behaves as a complex mixture, and even within the context of a homogenized model cannot be described by the classical Navier–Stokes fluid model. This is for at least two reasons. In narrow blood vessels, it is well known that blood shear thins (see discussion concerning the same later). In exceedingly narrow blood vessels, blood cannot even be modeled as a fluid, as the diameter of the blood vessel is comparable in size to the diameter of an erythrocyte.
- At times, one must deal with growth, adaptation, and remodeling that takes place in the blood vessel due to injury that it suffers. Most of the approaches to such problems use ideas such as “growth tensors,” etc., which have no proper biological and physical underpinning, merely mimicking techniques used in the mechanics of inelastic bodies that do not share a resemblance to growing, adapting, or remodeling biological materials.
- All processes that take place must comply with the demands placed by the Second Law of Thermodynamics, which states that the rate at which entropy is produced, ought to be non-negative. Even within the context of nonliving materials, one does not have agreement on how the Second Law ought to be interpreted (there are various interpretations of the Second Law of Thermodynamics due to Carnot, 7 Clausius, 8 Thomson, 9 Planck, 10 Caratheodory, 11 and others. An early attempt to develop a thermodynamic basis for living matter can be found in a treatise due to Schroedinger. 12

## Reasons why the Current Constitutive Models for the Aorta in Use Are Grossly Inadequate

Most, if not all, current models for the aorta assume that it is comprised of a nonlinearly elastic solid. In view of its walls being infused with blood carrying nutrients, such a description is untenable. Even if one was to accept the approximation that it is comprised of a nonlinearly elastic solid as being reasonable (and it is not), all these studies are hampered by not being able to clearly delineate what is called a stress-free reference configuration (a “baseline”), from which a deformation can be measured. All these studies that model the aorta, as being comprised of a nonlinearly elastic solid, employ the notion of a “deformation gradient,” which is invariably predicated on knowing a stress-free reference configuration. Also, one does not know if the reference state is undeformed (free of strain).

Much of the work that has been performed, assumes that the stress-free state of the aorta can be determined by repeatedly incising the aorta (either axially or transversely), based on an early study by Fung and coworkers (see Liu and Fung 13 and Fung et al 14 ;the existence of “residual-stresses” or “prestresses” in blood vessels was recognized much before the work of Fung and coworkers to determine them). There are several problems with such an approach, the least of which is the fact that the action of “cutting” is not what is referred to as a “diffeomorphism” in mathematics. That is, the mapping associated with the process of cutting is not differentiable. Moreover, one is never sure if all the stresses have been relieved after a finite number of cuts. Also, while such cutting leads to a global configuration, it does not guarantee that locally every point in the body under consideration is stress-free.

Such local problems aside, even the better current models in use to describe the aortic wall that take into account the difference in properties of the layers comprising the aorta, do not take into account the inhomogeneity within the layers. Inhomogeneity is patient specific, and hence it is exceedingly difficult to develop a model that is applicable to a general cross-section of patients. The specific nature of the inhomogeneity is critical with regard to catastrophic problems such as acute aortic dissection.

Continuum models to describe the response of bodies are essentially “homogenizations” that in an averaged sense reflect the constitution of the body. In the case of the aortic wall, underlying “homogenization” leads to a mathematical description of the material that is rendered exceedingly arduous by virtue of the body being comprised of a solid infused with a fluid-like material (blood), the latter in itself a very complex mixture that exhibits distinctly different response characteristics based on the nature of the vessel in which it is flowing.

### Which Is a Better Mathematical Description of the Aorta, a “Homogenized Viscoelastic Model” or a “Homogenized Mixture Model”?

The aortic wall is a mixture of solid and fluid constituents. The solid constituent of soft tissues is comprised of an extracellular matrix (e.g., collagen and elastin) and various cell types, while the fluid constituent comprises of blood (treated as a continuum), as well as intracellular and interstitial fluid. One could adopt two different approaches to modeling the response of the material of the aorta, the first is a “homogenized inhomogeneous viscoelastic model” or a “homogenized mixture model.” The latter approach is much more complicated, as it requires us to write at the very least balance equations for the mass and linear momentum for each of the constituents comprising the mixture. The theory supposes that the constituents that comprise the mixture can be homogenized over the domain of the mixture, so that at each point in the mixture, there is a particle belonging to each constituent, that is, the constituents cooccupy every point in the mixture (Truesdell, 15 16 Bowen, 17 and Rajagopal and Tao 18 ). Even if we were to simplify the problem by only considering the few constituents that are critical to the problem under consideration, the problem becomes exceedingly complicated. Moreover, when it comes to solving initial-boundary value problems within the context of mixture theory, one has fundamental difficulties with regard to the specification of boundary conditions. 18

**Fig. 2:**
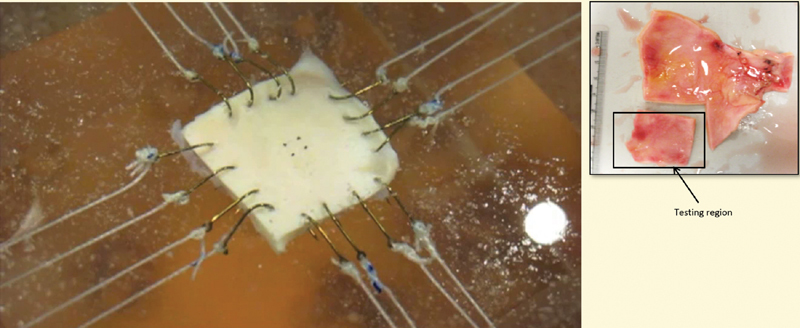
Biaxial system: a square specimen 20 mm × 20 mm with four hooks anchored on each tissue edge where suture lines extend from hooks to the motor arm and load cell. Two load cells for load measurements with four markers glued to the tissue surface for deformation measurements. Load and deformation measurements are synchronized (Private communication: power-point received from Prof. John Elefteriades).

While a rigorous mathematical procedure to carry out the homogenization to arrive at an equivalent inhomogeneous viscoelastic solid model for the aorta is not available, one can make an albeit crude phenomenological approach in developing a nonlinear inhomogeneous viscoelastic model guided by experimental data. However, to develop a reasonable model, we need to develop far more sophisticated experimental data than that which is available at the present moment. The best experiments that are performed at the moment are biaxial experiments ( Fig. 2 and **Appendix** ). However, to have models that have predictive capability with respect to clinical problems such as aortic dissection, we need to be able to carry out meaningful three-dimensional experiments, both with regard to gleaning the structure and morphology of the aortic wall, as well as the material response characteristics to various mechanical loading conditions.

**2. Modelling blood**

Blood is a complex mixture, consisting of gel-like cellular matter in a plasma solution. The cellular matter is comprised of erythrocytes, leukocytes, and platelets, and plasma is essentially a solution in which numerous proteins and various ions are dissolved in water as solvent. Even sans the numerous biochemical reactions that take place in flowing blood, keeping it in a delicate state of balance, the fact that one has so many constituents make the development of a constitutive relation to describe its behavior as a challenge. While plasma can be described by the Navier–Stokes constitutive theory, blood itself cannot be always described by the Navier–Stokes theory. While aortic blood flow can be approximated by the Navier–Stokes fluid model, its flow in the vasa vasorum cannot be. It has been well documented that blood shear thins significantly in narrow blood vessels (Charm and Kurland 19 and Chien et al 20 ) and stress-relaxes (Thurston 21 ). The shear-thinning of blood is attributed to the formation of erythrocyte rouleaux aggregates at low shear rates that are dismantled at high shear rates. The properties of blood change with structural changes at the microscopic level, a phenomenon referred to as thixotropy (Thurston 22 and McMillan et al 23 for measurements of the thixotropic characteristics of blood). With regard to the some of the other constituents of blood, the micropipette experiments of Evans and Hochmuth 24 indicate that erythrocytes exhibit viscoelastic behavior and those of Schmid-Schönbein et al 25 that leukocytes are also viscoelastic. Thus, numerous biochemical, physiologic, and rheological factors need to be taken into account to model the response of blood (see Anand et al 1 for a detailed discussion of the complex interplay between the various factors).

With regard to blood flow, previous studies either completely ignore biochemical reactions continuously occurring (e.g., the balance between coagulation and fibrinolysis under steady-state conditions) and merely incorporate fluid dynamic aspects, or on the other hand, completely ignore fluid dynamic issues, and focus on biochemical reactions under assumed conditions of static blood. For the purposes of this work, we are also guilty of such a gross simplification, focusing exclusively on mechanical considerations.

**3. Interaction between the flowing blood and the blood vessel wall**

Recently, a considerable amount of computational work has been performed that supposedly concerns itself with fluid-solid interactions that take place between flowing blood and the arterial wall. Some of these studies claim that they consider the whole circulatory system, arteries, arterioles, capillaries, etc., without recognizing that blood in extremely small vessels cannot even be modeled as a fluid. Such studies unfortunately give a totally false idea concerning the state of the art in cardiovascular medicine. In the absence of a proper model for the arterial wall, these efforts are at the best, a very crude portrayal of the actual phenomena, notwithstanding the bright, colorful, and stunning pictures that such efforts yield. Most of these studies assume that the arterial wall is a purely elastic body, at best some idealized simple nonlinear elastic model, blood being modeled invariably as a Navier–Stokes fluid. There are even attempts at modeling the whole circulatory system, but such studies are fraught with approximations that do not allow the problem under consideration to have any semblance of applicability to the real circulatory system.

### Applying Mechanics to the Study of Aortic Diseases

For over 500 years, the core functions of the cardiovascular system being mechanical in nature, and the phenotypes of cardiovascular diseases thus also being mechanical in nature, have been appreciated. Scientists from a very early date have applied the study of mechanics to the cardiovascular system (e.g., Leonardo da Vinci and Euler), and physicians have devoted themselves to the study of mechanics and even mathematics due to their desires to understand cardiovascular system functions (e.g., Harvey, Johann and Daniel Bernoulli, and Poiseuille). This is certainly true in the cases of aortic aneurysmal disease and dissection; Poiseuille's DSc thesis was in fact titled, “Recherches sur la force du coeur aortique.” However, as we have outlined, the mechanics required to describe and understand aortic diseases is complex. For these reasons, even state-of-the-art studies are not able to achieve the goals of modeling that we stated at the beginning of this article. So how should we proceed?

We suggest that it is essential to attempt to be as accurate, precise, and rigorous as possible with respect to the mathematics and mechanics used. Current aortic imaging techniques provide exquisitely fine detail with respect to local blood flow velocities, and aortic geometric features as functions of time (and thus by definition aortic deformation). However, because blood and aortic material properties are invariably uncharacterized, mischaracterized, or at best inferred based upon unjustifiable assumptions (Emerel et al 26 and Rajagopal et al 27 ), we do not have a good understanding of the forces exerted upon the aorta. But it is force that causes motion, and pathological loading conditions that contribute to aortic aneurysm and dissection. Deformation and other types of aortic motion are the ensuing result. Thus, instead of starting with high-fidelity geometry and measurements of aortic and blood motion, and attempting to back-calculate aortic loads (these are so called “inverse problems”), we suggest that it is important, despite how challenging it is, to attempt to discern aortic loads first, and then correlate these to aortic motion.

How would one accomplish such a goal in reality? The forces on the aortic wall are expressed in the stress tensor. Stresses are either normal, acting perpendicular to the aortic wall, or shear, acting parallel to the aortic wall. Normal stresses are related to blood pressure (mechanical pressure is the mean normal stress). However, knowing the pressure alone does not permit delineation of the individual normal stresses; aortic geometry and material properties must be determined. While the aorta and peripheral arteries do differ in terms of material properties, it is feasible to biopsy small portions of small arteries and measure their material properties; these could be extrapolated to aortic properties. Shear stresses on the aortic wall are caused due to the viscosity of flowing blood, which can be measured ex vivo, and blood velocity gradients that can be directly measured by imaging techniques (most notably magnetic resonance imaging). Thus, aortic motion can be measured, aortic and blood material properties can be measured, and aortic loads calculated with a minimum of assumptions. This is in contrast to current approaches, wherein aortic motion is measured, aortic and blood material properties are inferred, and aortic loads are calculated effectively based on several assumptions and circular reasoning. Finally, and perhaps most promising, microelectromechanical systems have been and are currently used to measure pressure, and could be configured to determine aortic stresses. This would result in the ideal situation, in which the disease-causative variables of aortic loads are measured, the resulting aortic motion also measured, and aortic and blood material properties can be calculated.

## Conclusions

A few final observations are in order. Most of the techniques and tools of continuum mechanics concern a body that has a fixed set of particles that respond to external stimuli. They are not meant to deal with their sudden appearance or disappearance, as in the birth and death of cells. These singular events also have associated with them the concepts of “life” and “death” that cannot be mathematically expressed and quantified. The theory of mixtures presents a very crude way of incorporating such ideas, but to be able to apply the theory of mixtures, one has to have a sufficiently “dense” set of each constituent, and this is not always the case with regard to biological multiconstituent matter. An elaborate discussion of these notions is beyond the scope of this article, but suffice it is to say that the state of the art in modeling biological materials is in its infancy, and such issues can be addressed at a much later date.

The resolution of problems in cardiovascular medicine and surgery has to be a team effort, with experts in medicine, biochemistry, mechanics, numerical analysis, electromagnetism, and other related fields, putting their heads together. However, unless there is overlapping expertise or some minimal knowledge in overlapping subsets of the above areas, conversations akin to those that took place in the tower of Babel will occur. The current state of affairs in the area leaves much to be desired, with investigators focused more in selling a bill of goods than the goods themselves.

## Notes

Appendix
**Anisotropy**
Initially defined with regard to light, “Acting in different ways to the way of polarized light; possessing the power of right-and left-handed polarization; (Oxford English Dictionary),” later adopted to response to mechanical forces.
**Biaxial Experiment**
An experiment in which planar bodies are tested by subjecting the specimen to deformations along two mutually perpendicular directions. One could either control the forces that are being applied or by the displacement along two mutually perpendicular directions (the control of the displacement is deceptive as the displacement is a consequence of the applied forces, that is, the forces have to adjust themselves to render the desired displacements).
**Differentiable**

A function *f* ( *x* ) of one variable *x* is said to be differentiable if a unique tangent can be drawn at every point of the curve *f* ( *x* )versus *x* . This idea can be generalized to a function of several variables.

**Inhomogeneity**
In mechanics, the notion of an inhomogeneous body is quite technical. A body is said to be homogeneous if there is some configuration in which the body can be placed in which all the properties of the body are the same at all the point belonging to the body. A body that is not homogeneous is said to be inhomogeneous. The fact that a body in a particular configuration has properties that are different at different points in the body does not necessarily mean that the body is inhomogeneous.
**Navier–Stokes Constitutive relation**

A precursor to the current Navier–Stokes constitutive relation was introduced by Navier 28 which had only one material constant describing a fluid, later Poisson 29 introduced a constitutive relation that is precisely the one currently used. Both Navier and Poisson used molecular arguments to develop the model. Stokes 30 developed the same model as Poisson on phenomenological grounds. Fluids which are described by the classical Navier–Stokes fluid model are also referred to as Newtonian fluids. This attribution to Newton is inappropriate for a variety of reasons (Dugas 31 and Truesdell 32 ).

**Non-Newtonian Fluids**
Fluids that cannot be described by the Navier–Stokes constitutive relation.
**Stress-Free Configuration**
A configuration of the body which in the absence of any boundary traction (forces) applied to it, is in a state of zero stress within the body.
**Thixotropy**
Time dependent change of the properties of a body. This might or might not be related to structural changes taking place in the fluid.
**Viscoelasticity**
Viscoelastic body refers to a body that is essentially an elastic body exhibiting viscous properties as opposed to an elasticoviscous fluid that is essentially a viscous body that also exhibits elastic properties. While viscoelastic fluids can exhibit stressrelaxation, an elasticoviscous fluid cannot exhibit stress relaxation.
